# Catalytic Asymmetric Amino Acid and Its Derivatives by Chiral Aldehyde Catalysis

**DOI:** 10.3389/fchem.2021.687817

**Published:** 2021-06-23

**Authors:** Kaijin Lin, Ang Shi, Chunhong Shi, Jinbiao Lin, Honggui Lin

**Affiliations:** School of Marine Engineering, Jimei University, Xiamen, China

**Keywords:** chiral aldehyde catalysis, amino acid, catalytic asymmetric, BINOL, substrate scope

## Abstract

Amine acid transformation is an important chemical process in biological systems. As a well-developed and acknowledged tool, chiral aldehyde catalysis provides good catalytic activation and stereoselective control abilities in the asymmetric reaction of N-unprotected amino acid esters and amino acid esters analogs, in which the key to success is the design of the catalysts derived from chiral BINOL aldehyde, which is based on the face control of enolate intermediates. In this review, one of the co-catalytic systems that combined with a transition metal to form a multiplex catalytic system and the well-established multiplex stereocenters of chiral aldehyde catalysis have been reviewed. Finally, a novel organocatalysis is prospected.

## Introduction

In the last decades, the use of aldehyde catalysis is one of the most important and prevalent transformations in organic chemistry (Li et al., 2018; Wang et al., 2019; Yabushita et al., 2019). It has attracted great attention for synthesis chemists to develop efficient aldehyde catalytic systems to mimic a biological process such as amino acid metabolism (Meng et al., 2018). The use of chiral aldehyde catalysis allows the enzyme mimicry and becomes an important synthesis tool in organic chemistry (MacDonald et al., 2011; Gong, 2019). It has become an efficient asymmetric synthesis strategy in amino acid chemistry (Su et al., 2018). The main chiral aldehyde catalysts contain chiral pyridoxal–dependent catalysis (Hoegl et al., 2018; Chen et al., 2019) and chiral binaphthol (BINOL) aldehyde catalysis (Li et al., 2017). Despite chiral pyridoxal analogs being developed and have successfully catalyzed asymmetric aldol condensation of amino acids, nowadays the stereoselectivity needs to be further improved and the development of this catalysis limits substrate activation. Therefore, highly desirable new organo-catalysts are developed to allow flexible and various substrates.

Except for the research works, some literature reviews have referred to these areas. For example, Subrata Shaw and James D. White summarized recent advances in asymmetric catalysis using chiral salen–metal complexes. It puts an emphasis on asymmetric phase-separative resolution of amino acids. Sun Li et al. reviewed new options for asymmetric organocatalytic reactions of aldehyde catalysis. They described Zhao and his coworkers developed a novel asymmetric Mannich reaction by introducing chiral aldehydes. This review summarizes articles covering the period up to 2018. Based on the previous review works, combining with the latest research articles, the aspects about chiral aldehyde catalysis that used transition-metal catalysis or asymmetric 1,6-conjugated addition and Mannich reactions are reviewed and summarized.

## Catalytic Asymmetric Amino Acid and Its Derivatives by Binary Chiral Aldehyde System

The asymmetric *α*-functionalization of primary amine and derivatives has an important impact in synthesis chemistry and is widely applied in the preparation of natural products and pharmaceuticals. Guo and his colleagues successfully synthesized a chiral BINOL aldehyde catalyst in 2014, which could directly catalyze the asymmetric *α*-functionalization reaction of the *α*-functional group of the N-unprotected amino ester (2-aminomalonate and A-alkylation of 3-indolyl methanol) *via* imine activation (Xu et al., 2014). In this organocatalytical system, the *α*-C-H bond of an amine can activate chiral BINOL aldehyde to form enamine through the corresponding 2-aza-allylic anion intermediates and deprotonation. Then the active imine carbon anion was formed and subsequently attacked electrophilic reagent. Finally, *α*-functional chiral primary amine was generated by the reversible *in situ* dissociation or amine exchange of imine. Although the enantioselectivity was improved by the utility of chiral BINOL aldehyde, the amino acid substrate could not be effectively extended, and the dosage of aldehyde catalyst is high. The development of efficient catalysts for amine acid transformation remains an elusive task. Inspired by this well-established enamine activation mode, synthetic chemists adopted this concept in the laboratory. Several biomimetic chiral BINOL aldehyde analogs or chiral pyridoxal analogs have been developed and employed in Mannich reactions (Dai et al., 2017), aldol reactions (Li et al., 2008), cyclization reaction (Itoh et al., 2015), and Cope-type hydroaminations of allylic amines (Guimond et al., 2012). In 2018, Zhao and coworkers designed N-quaternized pyridoxals as ideal enzyme mimics and successfully applied them in asymmetric Mannich reactions of glycinate with aryl N-diphenylphosphinyl imines (Chen et al., 2018). The catalyst exhibits high activity and stereoselectivity because of the bifunctional activation mode. It can activate not only glycine ester by the imine activation mode but also diphenylphosphine oxide–protected aromatic imines by hydrogen bond. In the same year, Guo and coworkers also reported a novel type of formyl chiral aldehyde derived from BINOL catalyst for the asymmetric activation of glycine esters through the asymmetric nucleophilic addition/cyclization reaction (Wen et al., 2018). Formyl chiral BINOL aldehyde served as the catalysts to directly activate the *α*-C–H bond of N-unprotected amines. It was based on the face control of the enolate intermediates. Chiral formyl BINOL aldehyde is an efficient nonpyridoxal-dependent catalyst that can promote the direct asymmetric *α*-functionalization of N-unprotected glycine esters. The successful usage of 2-formyl chiral BINOL aldehyde catalyst showed a high activity and stereoselectivity. It also expands the substrate including glycine esters, glycine amide, and glycine-derived dipeptides.

## Chiral Aldehyde Catalysis With Transition Metals

**FIGURE 1:**
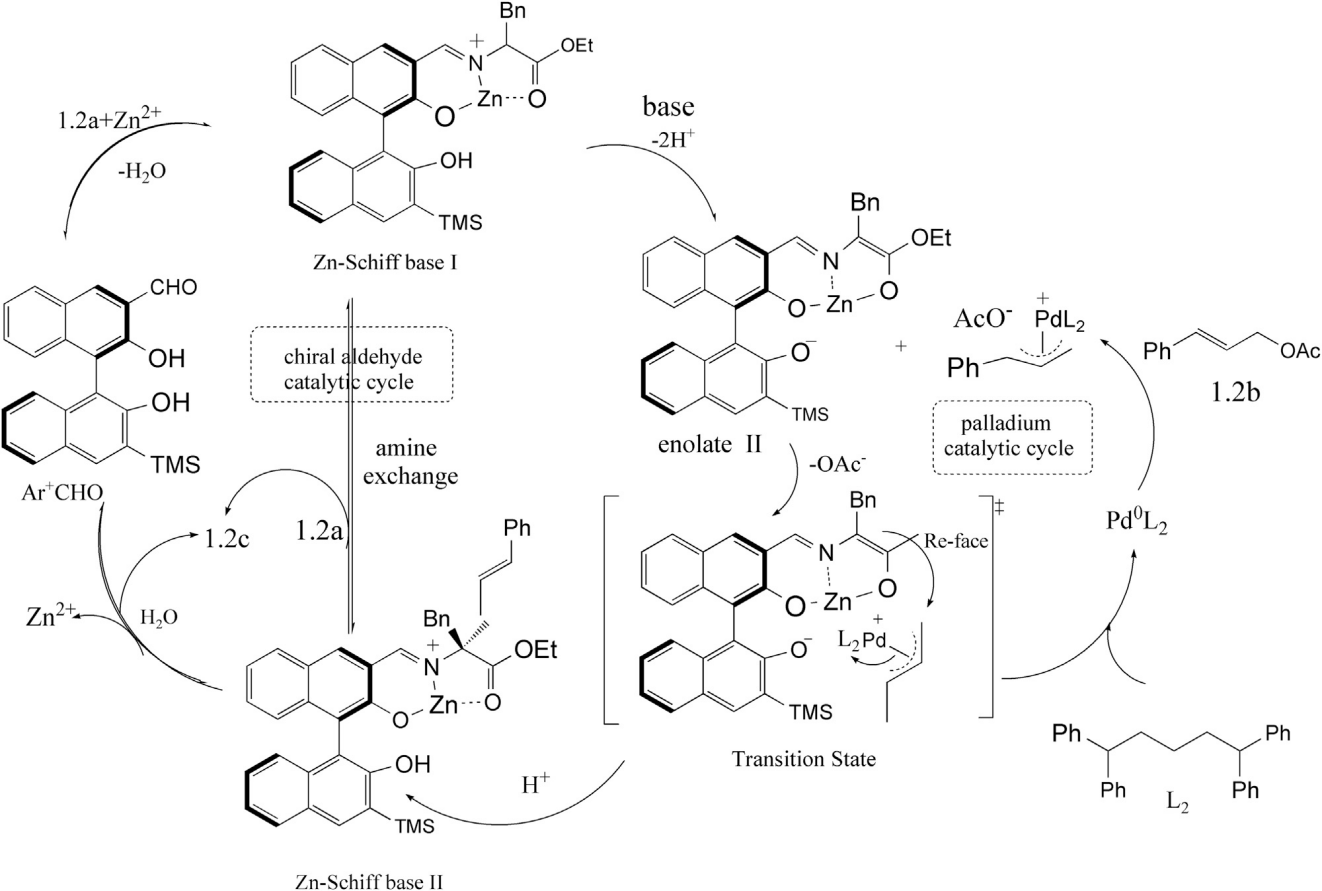
Combining chiral aldehyde catalysis and transition-metal catalysis for enantioselective *α*-allylic alkylation of amino acid esters (Chen et al., 2019).

Despite chiral aldehyde catalysis has become a powerful asymmetric synthesis strategy in amine chemistry, the development of catalysts for amine chemistry remains a great challenge. Inspired by a level of reactivity that could be achievable with the combinations of organic and transition-metal catalysts, Lei Chen and his coworkers provided a good strategy for the combination of chiral aldehyde catalysts with transition metals (Chen et al., 2019). In 2019, they provided a ternary catalytic system that is the combination of the Zn–Schiff base complex, chiral BINOL aldehyde, and Lewis acid (as shown in Figure 1). They successfully applied them in *α*-allylation reaction of N-unprotected amino acid esters and allyl acetates, exhibiting a good yield and enantioselectivity. With the expansion of the application of chiral aldehyde catalyst, new catalytic technologies are emerging. Guo and his coworkers have disclosed chiral 3-formyl BINOL aldehyde catalyst of anti–1,6-conjugate addition and syn-Mannich reaction that was inspired by the intermediate ketimine in transamination. Another chiral 2-formyl BINOL aldehyde catalyst gave the syn-selective conjugate addition and anti-Mannich products. The good yields obtained both the syn- and anti-products of transformations with high diastereo- and enantioselectivity.

**FIGURE 2:**
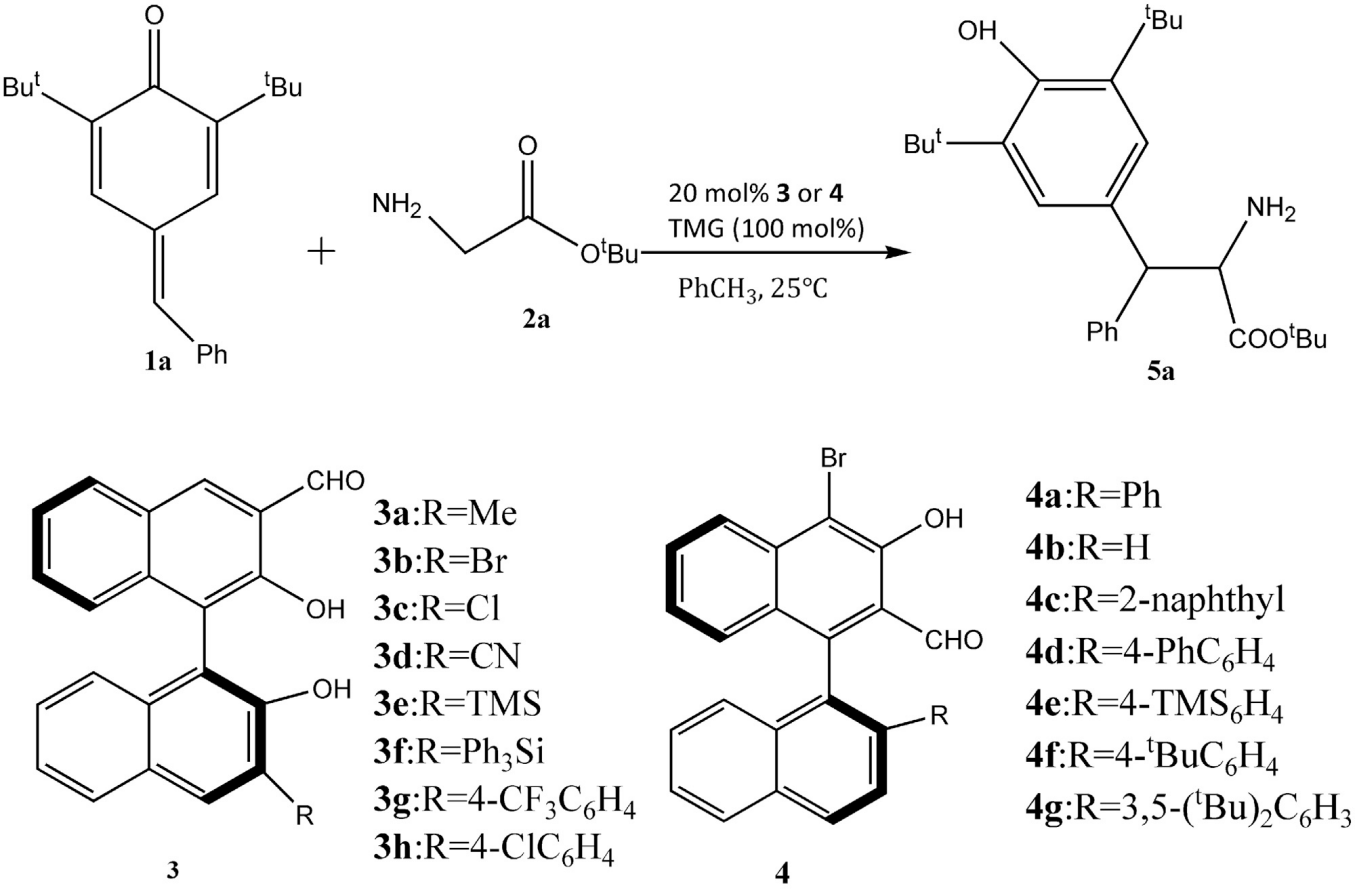
The 1,6-conjugated addition reaction by chiral BINOL aldehyde catalysts (Wen et al., 2020).

The novel chiral 3-formyl BINOL aldehyde catalyst was displayed in the study of the Mannich reaction of 1,6-conjugated addition of amino acids (as shown in Figure 2) (Wen et al., 2020). The reaction went well in the presence of 4g chiral aldehyde and mesitylene as the solvent and tBuOK as a base, leading to good yields with 90% ee with enantioselectivity. Notably, the catalyst loading was 10 mol%, and the reaction was completed after 7 h. Further studies showed that the substituted imines and pyridinylmethanamines in the syn-Mannich reaction could also be employed, although it required higher catalyst loading (20 mol%) and longer reaction time. Anti-product yields were affected by substituent steric and electronics. The developed catalytic system showed a quite broad substrate scope. A variety of para-quinone methide derivatives and amino acid substrates for the anti-selective reaction reacted well with chiral 3-formyl BINOL aldehyde catalyst to give the desired products in 25–84% yields with excellent enantioselectivities. However, it should be noted that when there is an imine bearing an electron-rich aryl substituent, the product yield decreased greatly. These indicated the aldehydes’ influence on the yield and formation of product and that electron-rich aldehydes worked well in this reaction. To demonstrate a stereoselective control, four potential reaction models were produced by DFT calculations. The key factor for the diastereoselectivity and enantioselectivity of catalysts is the steric effect of the R group. In the syn-Mannich reaction, phenyl imines bearing a single substituent on the phenyl ring leads to favorable reactivity, giving syn-products with good yields, diastereoselectivities, and enantioselectivities. Another key factor for the catalytic system is the strength of alkali. Because Schiff base intermediates are formed by alkali compounds, it was a critical factor for the nucleophilic attack to stereoselectivity. The demand for highly active electrophiles of chiral aldehyde catalysis strongly limits the application.

Chiral aldehyde catalysis promotes new methods to activate simple amines. However, no matter in the binary or ternary catalytic systems in asymmetric synthesis, the structurally diverse chiral aldehyde catalysts that were hardly achieved hampered the development of this field. Therefore, different aldehyde catalytic systems have been established. Professor Bing-Feng Shi has reported a system that synthesis of chiral aldehyde catalysts by Pd-catalyzed atroposelective C–H naphthylation (Liao et al., 2019; Liao et al., 2020). Jumpei Taguchi and his coworkers have synthetized potassium acyl trifluoroborates from aldehydes through copper (I)-catalyzed borylation/oxidation (Taguchi et al., 2019). Hirohisa Ohmiya et al. have gotten dialkyl ketones from aliphatic aldehydes through radical N-heterocyclic carbene catalysis (Yabushita et al., 2019; Kakeno et al., 2020). Sungwoo Hong group have employed an N-heterocyclic carbene (NHC) catalyst, which have developed a versatile catalytic system that enables deaminative cross-coupling reactions of aldehydes with redox-active pyridinium salts (Kim et al., 2020).

## Conclusion

In summary, the role of asymmetric catalysis by chiral aldehyde has grown rapidly in chemical reactions of amino acids. One limit is the reaction types. One more important limitation is how to get structurally diverse chiral aldehyde. Compared with binary catalytic systems, chiral aldehyde catalysts in ternary catalytic system not only acts as organic catalysts to activate amino acid and its derivatives through the formation of Schiff bases but also acts as ligands to promote the nucleophilic attack process. Importantly, compatibility also needs to be considered. Although the asymmetric catalysis involving chiral aldehyde has been grown in recent years, they are still rare. It is believed that the organic reactions catalyzed by chiral aldehydes are very important kind of asymmetric catalytic reactions, although some difficulties remain and new challenges will emerge. It is also believed that more researchers would involve and new and efficient chiral aldehyde catalysts will be developed.
